# Supplementary material for: Paternal Inheritance of Bisphenol A Cardiotoxic Effects: The Implications of Sperm Epigenome
Source: Int J Mol Sci. 2021 Feb 20;22(4):2125. doi: 10.3390/ijms22042125 (PMC7924642; doi:10.3390/ijms22042125)
Supplement: Supplementary file 1 [file ijms-22-02125-s001.pdf]

Analysis	Treatment	Test	Post-hoc test	p-value
***esr2a* expression**	100 μg/L BPA	ANOVA	DMS	0.090
	2000 μg/L BPA			0.837
***gper1* expression**	100 μg/L BPA	ANOVA	DMS	0.236
	2000 μg/L BPA			0.638
***esrrga* expression**	100 μg/L BPA	ANOVA	DMS	0.756
	2000 μg/L BPA			0.268
***esr2b* expression**	100 μg/L BPA	ANOVA	DMS	0.010
	2000 μg/L BPA			0.012
***hand2* expression**	100 μg/L BPA	ANOVA	DMS	0.821
	2000 μg/L BPA			0.045
***gata5* expression**	100 μg/L BPA	ANOVA	DMS	0.537
	2000 μg/L BPA			0.195
***gata4* expression**	100 μg/L BPA	Kruskal-wallis	Bonferroni correction	0.561
	2000 μg/L BPA			
***bmp4* expression**	100 μg/L BPA	ANOVA	DMS	0.063
	2000 μg/L BPA			0.094
***kat6a* acetylation**	100 μg/L BPA	Kruskal-wallis	Bonferroni correction	1
	2000 μg/L BPA			0.035
***esr2b* acetylation**	100 μg/L BPA	ANOVA	DMS	0.883
	2000 μg/L BPA			0.000
***hand2* acetylation**	100 μg/L BPA	ANOVA	DMS	0.803
	2000 μg/L BPA			0.013
***esr2b* methylation**	Control + 50 μM EGCG	Kruskal-wallis	Bonferroni correction	0.666
	2000 μg/L BPA			
	2000 μg/L BPA + 50 μM EGCG			
***hand2* methylation**	Control + 50 μM EGCG	ANOVA	DMS	0.570
	2000 μg/L BPA			0.535
	2000 μg/L BPA + 50 μM EGCG			0.570
**H3K9ac**	Control + 50 μM EGCG	ANOVA	DMS	0.649
	2000 μg/L BPA + 50 μM EGCG			0.422
**H3K27ac**	Control + 50 μM EGCG	ANOVA	DMS	0.726
	2000 μg/L BPA + 50 μM EGCG			0.563
***kat6a* acetylation**	Control + 50 μM EGCG	ANOVA	DMS	0.487
	2000 μg/L BPA + 50 μM EGCG			0.417
***esr2b* acetylation**	Control + 50 μM EGCG	ANOVA	DMS	0.786
	2000 μg/L BPA + 50 μM EGCG			0.118
***hand2* acetylation**	Control + 50 μM EGCG	ANOVA	DMS	0.822
	2000 μg/L BPA + 50 μM EGCG			0.290
***esr2b* expression**	Control + 50 μM EGCG	ANOVA	DMS	0.679
	2000 μg/L BPA + 50 μM EGCG			0.740
***hand2* expression**	Control + 50 μM EGCG	ANOVA	DMS	0.486
	2000 μg/L BPA + 50 μM EGCG			0.112
***kat6a* expression**	Control + 50 μM EGCG	ANOVA	DMS	0.267
	2000 μg/L BPA + 50 μM EGCG			0.343
**Embryo mortality at 24 hpf**	Control + 50 μM EGCG	ANOVA	DMS	0.773
	2000 μg/L BPA + 50 μM EGCG			0.300
**Embryo mortality at 48 hpf**	Control + 50 μM EGCG	ANOVA	DMS	0.506
	2000 μg/L BPA + 50 μM EGCG			0.202
**Embryo mortality at 72 hpf**	Control + 50 μM EGCG	ANOVA	DMS	0.520
	2000 μg/L BPA + 50 μM EGCG			0.189
**Embryo mortality at 96 hpf**	Control + 50 μM EGCG	ANOVA	DMS	0.687
	2000 μg/L BPA + 50 μM EGCG			0.097
**Embryo mortality at 120 hpf**	Control + 50 μM EGCG	ANOVA	DMS	0.667
	2000 μg/L BPA + 50 μM EGCG			0.114
**Cardiac malfor-mations**	Control + 50 μM EGCG	ANOVA	DMS	0.288
	2000 μg/L BPA + 50 μM EGCG			0.702

Figure S1. List of the different type of statistical analysis performed for each experiment, the post-hoc test used and the p-values obtained for each one.
